# Assessing Recent Efforts to Improve Organization of Cancer Care in Poland: What Does the Evidence Tell Us?

**DOI:** 10.3390/ijerph19159369

**Published:** 2022-07-30

**Authors:** Anna Sagan, Iwona Kowalska-Bobko, Małgorzata Gałązka-Sobotka, Tomasz Holecki, Adam Maciejczyk, Martin McKee

**Affiliations:** 1 European Observatory on Health Systems and Policies, London School of Economics and Political Science, London WC2A 2AE, UK; 2 European Observatory on Health Systems and Policies, London School of Hygiene & Tropical Medicine, London WC1E 7HT, UK; martin.mckee@lshtm.ac.uk; 3 Institute of Public Health, Faculty of Health Sciences, Jagiellonian University, 31-067 Krakow, Poland; iw.kowalska@uj.edu.pl; 4 Institute of Healthcare Management, Faculty of Economics and Management, Lazarski University, 02-662 Warsaw, Poland; m.galazka-sobotka@lazarski.edu.pl; 5 Department of Economic and Management in Healthcare, Faculty of Health Sciences in Bytom, University of Silesia in Katowice, 41-902 Bytom, Poland; tholecki@sum.edu.pl; 6 Lower Silesian Oncology Centre, 53-413 Wroclaw, Poland; adam.maciejczyk@dco.com.pl

**Keywords:** cancer care, healthcare, coordination, integrated care, Poland

## Abstract

Poland has implemented two major organizational changes in recent years to improve cancer care. In 2015, a dedicated ‘fast pathway’ to diagnostics and treatment was implemented for patients suspected of having cancer. In 2019, the National Oncology Network began pilots in four regions of care pathways for cancer at five sites. Neither has been evaluated—no baseline information was collected, and what assessments were undertaken were limited to process measures. While the 2019 initiative was at least piloted, a national rollout has been announced even while the pilot is still ongoing and when concerns about certain aspects of the model have been raised. Given that cancer is the second largest cause of death in Poland and that cancer outcomes are worse compared to Western European averages, there is a particular need to ensure that models of care are informed by the evidence and adapted to the realities of the Polish healthcare system.

## 1. Introduction

After cardiovascular disease, cancers are the second largest cause of death in Poland, accounting for 23% of all deaths in 2018 [1]. The most common cancer causes of death in 2020 were lung, breast, and colorectal cancers for women and lung, colorectal, and prostate cancers for men (https://ecis.jrc.ec.europa.eu/, accessed on 26 July 2022). Although the incidence of all these cancers is below the European Union (EU) average, death rates are higher (both age standardized) [1].

The explanations are, however, unclear, as cancer registration covers only about 10% of the population, and most published data on survival are now quite old [2]. However, the best available figures reveal lower 5-year survival rates, at 14% vs. 15% for the EU average for lung cancer, 77% vs. 82% for breast cancer, 53% vs. 60% for colon cancer, and 78% vs. 87% for prostate cancer [3]. There are national screening programs for breast, cervical, and colorectal cancers, introduced as part of the National Programme for Cancer Diseases Control for 2006–2015 [4] (later renewed for 2016-24 [5]), but a recent audit reported low participation (16%, 20%, and 40% of the respective target groups), with large geographical differences [6].

Historically, management of solid tumors in adults has been largely centralised on the Institute of Oncology in Warsaw and its two regional branches (in Gliwice and Krakow, both in southern Poland), but in reality, provision has been fragmented [6,7,8]. There are oncology centers in most of the 16 regions, which function either as autonomous stand-alone facilities or are part of general hospitals. Few, however, can provide comprehensive care. There are also non-academic cancer care centers, where medical students are trained, smaller satellite centers, and some oncology departments in other hospitals. Certain preventive, diagnostic, and therapeutic services, such as outpatient imaging diagnostics, inpatient clinical oncology, and radiotherapy, are also provided by private facilities [7].

Since the mid-2000s, several attempts have been made to improve health outcomes for cancer patients, including the adoption of the two National Programmes for Cancer Diseases Control mentioned above. However, as these programs made the implicit assumption that the organization of cancer care in Poland was largely appropriate, their focus was on strengthening preventative measures, including increasing participation in cancer screening programs and improving access to diagnostics and treatment, rather than on improving the organization of care. The closest that Polish policy has come to addressing this issue were the introduction, in January 2015, of a dedicated ‘fast pathway’ for patients with suspected cancer to enable them faster access to comprehensive diagnostics and treatment [9,10] and the piloting, from 2019, of the National Oncology Network, which is one of the pillars of the National Cancer Strategy 2020-30 [7].

In this perspective piece, we describe these two measures and assess whether they have contributed to improving cancer care in Poland. We begin by describing both policies in detail, including their implementation, followed by the problems they encountered before concluding with recommendations for moving forward.

## 2. Key Recent Reforms Aimed at Improving Cancer Care in Poland

### 2.1. Fast Pathway for Cancer Patients (2015)

A fast pathway for patients suspected of having cancer was introduced in 2015 as part of a package of measures to reduce waiting times for diagnostic investigations and specialist consultations [11]. The pathway covered all cancer sites. It involved, among others, expanding the roles of primary healthcare (PHC) physicians who were enabled to refer, within guidelines, to this fast track, introducing maximum waiting times for diagnosis and treatment, and abolishing financing limits on coverage of services provided within the pathway. The latter led to a substantial increase in the total spending on cancer care, including on innovative pharmaceuticals, from PLN 7.6 billion (€1.6 billion) in 2014 to PLN 10.4 billion (€2.2 billion) in 2019—an increase of over 35% over 5 years [12].

The pathway starts with a visit to a primary care or outpatient specialist doctor who assesses the patient and—if a cancer diagnosis is suspected—issues the patient with a Diagnostics and Oncology Treatment (DiLO) card. This card entitles the patient to fast-track access to necessary diagnostics and then to treatment within guaranteed maximum waiting times: 28 days from the visit to the primary care doctor to basic diagnostics, 21 days from the specialist consultation to in-depth diagnostics, and 14 days from the multidisciplinary Concilium (Box 1) to the start of treatment. The targeted maximum waiting times for basic and in-depth diagnostics were not to have exceeded 9 weeks in 2015, 8 weeks in 2016, and 7 weeks from 2017. If the DiLO card is not issued, the patient enters—next to all other patients—general waiting lists for diagnostic tests and treatment.

Box 1 **The multidisciplinary concilium.**

The concept of a multidisciplinary concilium was created as a means to provide integrated high-quality cancer care appropriate to the patient’s needs. It comprises a clinical oncologist, radiotherapy specialist, oncological surgeon, and (for blood cancers) a hematologist. It meets once the initial diagnostic phase has been completed. Other specialists may also be included, depending on the type of cancer, and further support may be provided by other health professionals, such as nurses, psychologists, physiotherapists, and clinical geneticists. It also includes a care coordinator who supports the patient when making appointments, ensures the flow of information among the various healthcare providers and with the patient, manages their medical records and their DiLO card, and liaises with their primary care or specialist outpatient provider on discharge.

Source: Based on [1].

Even before it was enacted by Parliament, this ‘oncology package’ faced criticism from healthcare providers and those commenting on health policy [11]. They were concerned that it was to be implemented across the entire country without being piloted, and without involvement of primary care doctors, who despite their critical role in the implementation of the pathway were simply presented with new contractual obligations as a fait accompli and faced termination of contracts if they rejected them [13].

Once the implementation had started, operational shortcomings quickly became apparent, although some were addressed in subsequent legal amendments [14]. For example, since many oncological diagnostic tests, ranging from blood tests to endoscopies, had previously been available only to ambulatory specialists, primary care doctors required training in the indications for ordering them, and the interpretation of results and their competencies had to be expanded to allow them to order such tests. Furthermore, initially, only primary care doctors could issue the DiLO cards, so patients with cancers diagnosed by specialists still had to see their primary care doctors to access the new pathway, with the inevitable delay in starting treatment. The remaining shortcomings are summarized in Table 1.

It Is difficult to say whether the reform led to any improvement in terms of waiting times or enabled earlier diagnosis. For example, only 17.4% of basic diagnostics and 7.9% of in-depth diagnostics were completed within the maximum waiting limits in 2016 [6], although these shares appear to have increased to about three-quarters between 2018 and 2020 [19]. The total waiting times for patients included in the pathway seem to have shortened during the first 1.5 years compared to patients whose cancers fell outside it [6], with some evidence that it may even have lengthened for the latter group [6,20]. However, it is difficult to be certain about what has happened, as providers can easily ‘play the system’ by registering patients only after they have been admitted for diagnostics or treatment rather than when the cancer was first suspected. In addition, there was no reliable information about waiting times before the fast pathway was introduced, and thus no baseline against which the new pathway could be assessed.

There are also difficulties in assessing progress toward a greater role for PHC, as opposed to specialist ambulatory care. Only about a third of referrals to the fast pathway came from PHC in 2015 [6], a figure unchanged by 2018–2020 when the number of referrals was much higher [19], as there was a corresponding increase in referrals from ambulatory specialists [20]. 48% of patients who were referred to the pathway were found to have cancer, and this has been interpreted as indicating a high threshold for referral, with patients whose symptoms were less specific not being referred [6]. It has also been noted that referral rates differ enormously geographically (up to 280%) in ways that do not correlate with cancer incidence [6]. Several reasons have been suggested, including insufficient training of PHC doctors in cancer detection, in part reflecting the lack of financing for such training or for diagnostic tests (see Table 1), but also fears of financial sanctions if the share of confirmed cases in patients with cancer suspicion is lower than a certain threshold. Interestingly, during the COVID-19 pandemic, PHC saw the smallest decrease in the number of issued referrals, which was ascribed to the higher use of teleconsultations and increased oncological vigilance [21].

An unintended consequence of the introduction of maximum waiting times was the increased fragmentation of cancer care [6,17]. This was already a problem in 2012, when 806 hospitals provided oncology services [22]. By 2017, two years after the reform, 2600 providers were contracted to provide services within the oncology pathway, but only about 18 of them (less than 1%) provided over 50% of the contracted services [17]. In 2015, only 28% of hospitals and 2% of specialist ambulatory providers participating in the oncological package could provide laboratory tests, CT and MRI scans, and endoscopy examinations, and fewer than half of audited providers could undertake intraoperative pathology necessary to assess margins of some tumor excisions [6]. This last problem has been commented on in a recent report by the National Audit Office [23]. There have also been concerns about the lack of standardized diagnostic and treatment pathways in cancer care and the quality of cancer provided in some facilities, especially those with low volumes of care [9,16,24]. In 2020, only 18 providers reached recommended thresholds for the number of treated patients with lung cancer: 38 for colorectal cancer, 11 for breast cancer, and 21 for prostate cancer [25].

These concerns have been around for some time. A national audit covering the period from January 2015 to May 2016 reported wide variations in the quality of cancer care among providers [6,22]. For example, some hospitals lacked access to modern immunohistochemical and molecular diagnostics. Many providers participating in the fast oncology pathway, especially smaller single-specialty centers with fewer specialists, faced practical problems in convening the multidisciplinary conciliums prescribed in the pathway, and as noted in the 2017 national audit, had to collect specialists from larger multispecialty centers by bus (a phenomenon that even had a name, a ‘bus concilium’) [6]. Anecdotal evidence suggests that, in some cases, only signatures were collected, and the conciliums did not actually take place [26].

### 2.2. Pilot of the National Oncology Network (2019–2022)

The shortcomings described above resulted in new proposals for improving cancer care and culminated in the adoption of the National Cancer Strategy 2020-30 [7], which replaced the National Programme for Cancer Diseases Control for 2016–2024. This is aligned with Europe’s Beating Cancer Plan [27]. The strategy supports the introduction of the National Oncological Network as a way to improve organization of the cancer care system while at the same time strengthening investment in primary and secondary prevention (including within the Network), in human resources, science, and innovation.

The concept of this Network draws on experiences from other countries in Europe, in particular Norway, France, and the United Kingdom [17]. It seeks to improve primary and secondary prevention, early diagnosis, and quality of treatment for all patients, irrespective of their place of residence. It envisages standardization of care pathways, concentration of expertise in highly specialized procedures, and quality monitoring. The Network groups existing public and private providers of cancer care into three reference levels, each with specified competencies and principles for cooperation. These providers are meant to provide comprehensive and coordinated cancer services, covering primary and secondary prevention, diagnostics, ambulatory and hospital treatment, post-treatment monitoring, rehabilitation, palliative care, and hospice care. The new structure is expected to ensure that none of these elements are missed, and that each is carried out according to strictly defined standards.

**Figure 1:**
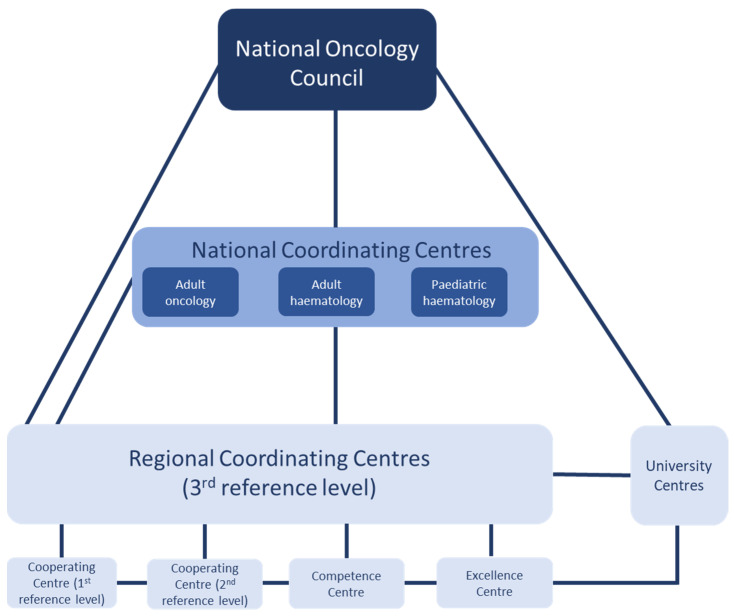
Proposed organization of the National Oncology Network, developed in 2018. Source: Authors based on [17] (p. 38).

The activities of the Network are to be coordinated and managed by the National Oncology Council (Figure 1). The Council will be responsible for setting standards and accrediting providers included in the Network, as well as for monitoring the overall functioning of the system. Three National Coordinating Centres, in adult oncology, adult hematology, and pediatric hematology will be responsible for developing and updating diagnostic and therapeutic guidelines, for professional training, initiating research (in collaboration with university centers), as well as for health promotion and cancer prevention activities, in their respective areas of expertise.

Allocation to one of the three levels is based on resources and volume of activity and, for the two lower levels, the demonstration of formal cooperation mechanisms with higher levels. Those at the 3rd reference level will function as the Regional Coordinating Centres, responsible for deciding where each patient will receive treatment, depending on their site and stage of cancer. Providers with expertise in a particular area can qualify as competence or excellence centers (e.g., a breast cancer unit). In addition, local cooperating or satellite centers (local hospital departments or ambulatory clinics) will provide services such as day-case chemotherapy, post-treatment monitoring and care, and rehabilitation. This is to enable care to be provided close to patients’ homes, consistent with developments in countries such as Norway and the UK [28]. These providers will cooperate with PHC teams, offering the first contact point for specialist consultations as before, but with closer coordination. The Network will provide training and support for patient pathways [17]. Providers outside the Network will no longer receive public financing for cancer services.

Since early 2019, this model has been piloted in two regions (Dolnośląskie in the southwest and Świętokrzyskie in south-central Poland), with one more region joining in October that year (Podlaskie in the northeast) and a further one in April 2020 (Pomorskie in the north) [29]. The aim of the pilot was to test the new model, create a data collection system to monitor treatment results, adverse events, and complications, and analyze them by area of care, region, and at the level of individual providers [17]. This was preceded by the development of patient pathways, with diagnostic and therapeutic guidelines established for the five most common cancers (lung, colorectum, breast, prostate, and ovary) and the selection of metrics and indicators. The pilot was expected to show improvements in the quality and safety of cancer treatment, patient satisfaction, and cost-effectiveness [17].

Providers participating in the pilot implemented standardized patient pathways for these five cancers, with standardized protocols and documentation. Standardized documentation was also introduced for pathology and radiological findings, with standardized templates for patient data to be used in multidisciplinary conciliums, which for the first time in Poland includes information about the stage of cancer. Quality monitoring covers the timeliness of diagnostic tests and treatment, completeness of diagnostics, and 35 quality indicators [30] (see Table A1 in Appendix A), which can be compared with other centers participating in the pilot and with international data.

Care coordinators, previously used only during the treatment phase, were brought in from the start of the patient’s journey. Their responsibilities were formalized, with defined responsibilities and procedures for working with medical staff, including standardized checklists to ensure that all the necessary data were gathered before the concilium, and access to IT support in monitoring patient progress along the pathway. Dedicated call centers were introduced to provide information about cancer care. Patient satisfaction was monitored with a survey that asked about timeliness, complexity, ease of access, and general experience of care.

The pilot was originally meant to run for 1.5 years until the end of 2020. This was extended until the end of 2022 due to the COVID-19 pandemic but also due to problems interpreting the results. Official data published at the end of 2021 show mixed results across the four regions (see Table A2 in Appendix A), with positive appraisals of the pilot mainly from the Lower Silesia (Dolnośląskie) Oncology Centre [31,32]. This is likely related to what was already high quality of care provided by the Centre, which independently of the pilot was the first cancer center in Poland to be awarded the International Innovative Partnership for Action Against Cancer (iPAAC) certificate. Even there, the evaluations focus on organizational changes, such as implementation of standards, and basic process measures, such as numbers of patients included and calls to the Infoline) rather than on indicators of quality, access, and patient outcomes. This is partly because it is too early to see improvements in patient outcomes after such a short time, but also because similar information is not collected with respect to patients not participating in the pilot or lack of baseline indicators. Assessment has been further complicated by the COVID-19 pandemic, which has affected the provision of cancer screening, diagnostics, and curative services [21]. Thus, at this point, an objective assessment of the pilots is difficult, if not impossible.

## 3. Discussion

On 4 February 2021, on World Cancer Day, the Polish government announced that it would prepare a draft law on the National Oncology Network by the end of March that year to be implemented on 1 January 2022, effectively rolling out pilots across the whole country. Indeed, despite the lack of any final assessment of the pilot, the draft was published and submitted for public consultation. This was based on a preliminary assessment of the pilot prepared by the Polish Cancer Society, but that report has so far not been published for public scrutiny. While many of the changes tested in the pilot are welcome and have the potential to significantly improve the quality of cancer care in Poland, the draft law has also attracted criticism.

One source of concern is that the pilot, and now the draft law, focuses mainly on hospital care, neglecting preventive activities and the early detection of cancers, despite concerns that many cancers are detected too late to be treatable. While the new cancer units are meant to provide preventive services alongside diagnostics and treatment, this is no substitute for community prevention and is unlikely to have a wide-enough reach. Cancer prevention could be more effective if it were provided within PHC, but the Regional Coordinating Centres lack any means to influence the provision of services at the PHC level. It is also unclear if this would have made any difference, as PHC providers in Poland are generally not very effective in providing preventive services, and recent plans to strengthen preventive services at the PHC level [33] have been recently scrapped. Other services, such as oncological rehabilitation, palliative care, and hospice care, are also largely neglected in the draft, however, they are crucial elements of comprehensive cancer care.

Other commentators are concerned that the new model may be too difficult to implement in the Polish context. For example, some analysts argue that the uneven geographical distribution of human and infrastructure resources makes it impossible to establish reference facilities in all regions. For example, surgery for lung cancer is only provided in a few centers. The exclusion of providers not meeting the quality criteria for the Network may reduce fragmentation of care and improve quality, but creates problems for patients currently served by these providers, who may face longer journeys. A certain degree of flexibility may thus be needed to adapt the model to the current circumstances, at least initially. While the draft law gives providers 2 years to implement the new quality requirements, this may not be long enough to establish reference facilities in all regions.

The 2015 reform that introduced the fast cancer pathway also failed to adequately consider the realities of the Polish healthcare system. One example is the intention to increase the role of primary care in cancer diagnosis, which faces the challenge of an already high burden falling on PHC doctors and associated financial and staff shortages [13,33]. The organizational problems that emerged after the reform was implemented, without a proper pilot, and the unforeseen negative effects of the reform, such as the increased fragmentation of cancer care provision described above, provide a warning that ill-designed reforms may not only be hard to implement but also create new problems.

The piloting of the National Oncology Network offers the opportunity to introduce necessary fixes before the Network is implemented across the whole country. Rushing in with the legislation while the pilot is still underway, without a thorough assessment or addressing concerns that have already surfaced, defeats the aim of having a pilot in the first place and risks introducing mistakes on a larger scale.

However, as mentioned earlier, a thorough assessment of the pilot may be difficult. There were no baseline data and little comparative data outside of the pilot. This highlights the broader problem of the lack of data to underpin decision making in the Polish healthcare system. Reliable and sufficiently granular data on the incidence, stage of cancer progression, treatments, and waiting times for various services are largely missing. Some information is collected in the National Cancer Registry, but as noted above, coverage is limited and often delayed. Providers focus instead on reporting process information on services necessary for financial settlements with the public payer—the National Health Fund (NHF). Such data are then used to construct the maps of health needs (which were introduced in 2016 as a decision support tool), including cancer care. This creates a bias toward the existing structure of service provision and distribution of resources, such as the focus on hospitals, diverting attention from the relatively less well-developed but much-needed outpatient services and prevention [27,34]. Since the draft law focuses on hospital care, it may further entrench this imbalance in the provision of cancer services. The inertia is reinforced by the NHF. For example, although the maps suggested improving the concentration of invasive treatment by cutting the number of contracts in general oncological surgery from 332 in 2015 to 260 in 2018, as many as 327 contracts were still issued by the NHF in 2018—a meager fall of 1.5% instead of the desired 22% [28].

Metrics and indicators collected within the National Oncology Network represent the beginnings of the first system of quality assessment of cancer care in Poland that can support evaluations at the national, regional, and provider levels. This is a welcome development, and it may help drive real change and a gradual departure from the status quo.

Despite all their shortcomings, the testing of reforms via pilots, such as through the National Oncology Network pilot, is a welcome development in Polish health policy. It was only in 2017 that a legal amendment [35] introduced pilot programs in the health system as a means of testing new ideas before introducing them at the system level [36]. This is certainly a positive change, but experience shows that it requires much finer tuning, both in designing the pilots and learning from them before initiating country-wide rollouts. Pilots would likely benefit from increased stakeholder engagement to ensure that proposals can be implemented in practice. This is, to some extent, achieved through public consultations on draft legislation, to which all legal acts are mandatorily subject, but these are not always carried out appropriately. For example, public consultations that preceded the implementation of the fast cancer pathway were too short to allow for the meaningful involvement of all stakeholders [6]. Opaque decision making can further erode trust in the reform and undermine implementation. The fact that the draft law on the National Oncology Network was based on a report that only a handful of people at the Health Ministry had access to has raised many questions about the results of the pilot—these doubts could have been cleared and possibly addressed if the report was made public.

## 4. Conclusions

Cancer is a major health problem afflicting the Polish population. Improving cancer outcomes by better prevention, detection, treatment, and post-treatment services are of paramount importance. Given the various problems with the provision of cancer services in Poland, reforms are much needed. The measures assessed in the National Oncology Network pilot, such as the introduction of reference levels, strengthening of care coordination, introduction of standard patient pathways for diagnostics and treatment, comprehensive monitoring of quality indicators, and many others, hold promise. However, they must be carefully designed, adapted to the needs and reality of the Polish healthcare system, and carefully and transparently evaluated. For example, focusing attention on hospital treatment and neglecting prevention will not lead to improved health outcomes if cancers continue to be detected too late for treatment to be effective. Additionally, basing decisions on one pilot site, which is already known to deliver high-quality cancer care even before the introduction of the pilot, dismisses the realities in the rest of the country, where resources and quality of care are worse and where the piloted solutions may not be implementable. A lack of transparency about the results makes it difficult to optimize proposed solutions and risks introducing mistakes that will have real consequences for the patients.

As in other countries, politics exerts considerable pressure on Polish health policy. There is thus pressure to keep the promises made on 2021 World Cancer Day before definitive evidence on the effects of the pilot becomes available. This should be avoided. Doing something just for the sake of it may waste already scarce resources without bringing much value to cancer patients. All indicators collected in the pilots should be published and subjected to public debate, allowing experts, patients, and other stakeholders to work out joint solutions—either in the form of revisions to the proposed draft law on the National Oncology Network or otherwise—that can be effectively implemented in all regions. These should focus not only on hospital treatments but encompass the entire patient pathway, as well as health promotion and disease prevention activities, and not only the current configuration of resources but also how to improve them going forward. The new National Cancer Strategy addresses many of these concerns, and policymakers should focus on implementing all its elements, not only the National Oncology Network, while fine-tuning the latter. After little progress has been made in improving detection and treatment pathways over the past few years, Polish patients deserve better.

## Figures and Tables

**Table 1:** Key measures introduced in the fast oncology pathway and their main shortcomings.

Measures	Key Shortcomings
**Fast access to diagnostics and treatment**	
PHC doctors were tasked with issuing DiLO cards for patients with suspected cancer, which give them fast access to diagnostics and—if the cancer suspicion has been confirmed—to treatment	PHC doctors did not receive any additional training (or funding to finance such training) in cancer detection No additional financing was provided to cover the costs of basic diagnostics (these had to be covered within the existing capitation rates) Advanced diagnostics, such as CT or MRI scans, which are needed to detect some cancers, can only be ordered by specialist doctors [15]
Maximum waiting times for basic and in-depth diagnostics and treatment were introduced, with financial incentives for providers to observe them (penalties up to 30% of the value of contracted services)	There are no maximum waiting times for the entire pathway There are no standardized guidelines for diagnostics and treatment No single provider is responsible for the entire pathway There is no comprehensive, standardized evaluation of the quality of cancer care and health effects of applied treatments [9]
**Comprehensiveness of diagnostics and treatment**	
Introduction of a multidisciplinary concilium charged with planning the course of treatment	Fragmentation of care means that providers face practical problems in gathering together the conciliums Participation of a radiologist in the concilium has been made optional since 2017 [16]
Introduction of a treatment coordinator charged with supporting the patient on their treatment pathway	No coordination support is available during the diagnostic phase There are no uniform guidelines regarding the role and tasks performed by the coordinators (and no uniform training)
Abolishment of the financing limits for services covered within the pathway	Valuation (prices) of some of the services contracted within the pathway was reduced [17] The pathway does not cover all cancers ^a^, all types of patients ^b^, settings where patients may be diagnosed ^c^, and services ^d,e^ [18] The pathway does not include post-treatment follow-up and prophylaxis [18]

Notes: ^a^ e.g., cancers that are not diagnosed with a histopathological examination, including testicular, kidney and adrenal cancers; skin cancers (except for melanoma) and sarcomas in adults. ^b^ e.g., patients with two cancers and patients with a relapse. ^c^ e.g., patients diagnosed in emergency departments. ^d^ e.g., Positron Emission Tomography (PET) scans, psychological support, palliative care services, enteral and parenteral nutrition, and blood transfusions. ^e^ Access to the latest therapies is limited in Poland; for example, only 53% of modern oncological drugs authorized for use in Europe are available [16]. DiLO card = Diagnostics and Oncology Treatment card; CT = computerized tomography; MRI = magnetic resonance imaging. Source: Authors.

## Data Availability

Not applicable.

## Appendix A

**Table A1:** Quality indicators monitored in the National Oncology Network pilots, 2019–2022.

	Indicator
1	Percentage of deaths within one year from the diagnosis of malignant neoplasm, broken down by cancer stage
2	Percentage of deaths within 30 days from the date of surgery, broken down by cancer stage
3	Percentage of deaths within 30 days from the end of chemotherapy, broken down by cancer stage
4	Percentage of deaths within 30 days from the end of palliative radiotherapy, broken down by stage cancer
5	Percentage of patients requiring hospitalization due to complications after surgical treatment
6	Percentage of patients requiring hospitalization due to complications after radiotherapy
7	Percentage of patients requiring hospitalization due to complications after systemic treatment
8	Percentage of patients who received chemotherapy as inpatients
9	Percentage of patients with stage III and IV of cancer
10	Assessment of the completeness of pathological examination
11	Percentage of patients who were tested for genetic and molecular predictors
12	Percentage of surgical procedures performed with minimally invasive methods
13	Median time that has elapsed from the date the patient was issued a referral for a diagnostic (imaging or pathomorphological) examination to the date of obtaining the result of this examination
14	Percentage of diagnostic tests repeated within 6 weeks (computed tomography, endoscopy, biopsy, pathomorphological assessment, molecular assessment), by provider, type of tumour and type of examination
15	Percentage of repeated treatments in diagnoses other than breast cancer
16	Percentage of patients with rectal cancer who received preoperative radiotherapy
17	Percentage of postoperative histopathological examinations in patients with colorectal cancer in which the number of assessed lymph nodes was at least 12
18	Percentage of patients with colon and rectal cancer with anastomotic leakage
19	Assessment of the number of lymph nodes removed during prostatectomy
20	Percentage of pelvic lymphadenectomy performed with the division of histopathological material according to anatomical ranges
21	Number of positive postoperative margins after prostatectomy
22	Percentage of patients with suspected lung cancer consulted by a pulmonologist within 14 working days from the date of registration of the referral with the service provider
23	Percentage of patients with mediastinal lymphadenopathy greater than 10 mm, who underwent EBUS-TBNA
24	Percentage of patients with suspected lung cancer and pleural effusion, with diagnosed fluid aetiology
25	Percentage of patients with stage III non-small cell lung cancer who received simultaneous chemoradiotherapy
26	Percentage of patients with ovarian cancer treated with primary optimal or suboptimal cytoreduction (leaving no residual mass or <1 cm)
27	Percentage of patients with ovarian cancer who received neoadjuvant chemotherapy (NACT)
28	Percentage of patients with ovarian cancer who underwent exploratory laparotomy
29	Percentage of patients with non-infiltrating neoplasm with a diameter of less than 2 cm (after excluding patients with BRCA1 and BRCA2 mutations) undergoing breast-sparing treatment
30	Percentage of patients with infiltrating neoplasm with a diameter not exceeding 3 cm (total size, including DCIS component; after excluding patients with BRCA1 and BRCA2 mutations) undergoing breast-sparing treatment
31	Percentage of patients with non-infiltrating neoplasm with a diameter of not more than 2 cm (after excluding patients with BRCA1 and BRCA2 mutations) undergoing breast-sparing treatment
32	Percentage of DCIS patients who have not had the contents of the armpit removed
33	Percentage of patients with infiltrating cancer without lymph node metastases (pN0), in whom the lymphatic system of the armpit was not removed
34	Percentage of patients with hormone-sensitive infiltrating cancer who received hormonal treatment
35	Percentage of patients with inflammatory neoplasm or locally advanced, unresectable ER-expressing breast cancer who underwent induction chemotherapy

Source: [30].

**Table A2:** Selected results of the National Oncology Network pilots in the four regions, change between June 2020 and June 2021.

Region (Number of Included Patients *)	Median Time from Registration for Diagnostic to Obtaining Results	Percentage of Patients with Genetic and Molecular Tests	Percentage of Patients Who Needed to Be Hospitalised Du to Post-Surgery Complications
Dolnośląskie (11,688)	↓ Decrease from 19 to 13 days	→ Unchanged at 95%	↓ from 3% to 2%
Podlaskie (1827)	↑ Increase from 6 to 8 days	↑ Increase from 75% to 97%	↓ from 3% to 2%
Pomorskie (3025)	↓ Decrease from 18 to 11 days	n.a.	↓ from 10% to 7%
Świętokrzyskie (3984)	↓ Decrease from 13 to 12 days	↓ Decrease from 100% to 92%	↓ from 3% to 2%

Note: * Number of patients included in the pilots as of 30 June 2021. Source: Authors based on [29].
